# Persistent Idiopathic Facial Pain Associated with Somatoform Disorder in an 11-Year-Old Boy

**DOI:** 10.1155/2019/4627850

**Published:** 2019-02-07

**Authors:** Yoshihiko Sakurai, Asami Fujii, Fumie Kato

**Affiliations:** ^1^Department of Pediatrics, Matsubara Tokushukai Hospital, 7-13-26 Amami-higashi, Matsubara, Osaka 580-0032, Japan; ^2^Department of Psychosomatic Medicine, Matsubara Tokushukai Hospital, 7-13-26 Amami-higashi, Matsubara, Osaka 580-0032, Japan; ^3^Palliative Care Center, Nara Medical University Hospital, Kashihara, Nara 634-8522, Japan; ^4^Department of Psychosomatic and General Internal Medicine, Kansai Medical University, 2-5-1 Shinmachi, Hirakata, Osaka, Japan

## Abstract

Persistent idiopathic facial pain (PIFP) is a poorly understood chronic disorder that rarely occurs in children. An 11-year-old boy initially presented with right cheek pain and a streptococcal infection 6 weeks previously. Facial cellulitis was suspected, which was resolved by antibiotic treatment. The right cheek pain recurred within 4 weeks of this initial visit. Because the antibiotic treatment did not relieve the pain, the patient visited our outpatient clinic. Physical examination revealed facial tenderness in an area that corresponded with the region supplied by the second branch of the trigeminal nerve (maxillary nerve), suggesting trigeminal neuralgia (TN). However, brain magnetic resonance imaging revealed no vascular compression. Furthermore, the continuous nagging and dull nature of the pain experienced by the patient differed from the sudden and severe nature of pain associated with TN. Subsequently, PIFP was diagnosed. The patient was unable to attend school because of prolonged lassitude, nausea, headache, and anorexia. Psychological counseling revealed psychological stress related to his out-of-school life. Upon learning stress management through psychotherapy, his general malaise gradually improved, and he was able to attend school with more facial expressions. This case indicates the psychogenic aspect of PIFP as well as the value of psychological counseling.

## 1. Introduction

Chronic pain is often seen during childhood. Thirty percent of children, including adolescents, have chronic pain that persists for more than 3 months [1]. Furthermore, more than 50% of children between the ages of 9 and 13 years experience chronic pain at least once [2]. Headaches, abdominal pain, and musculoskeletal pain are the major types of chronic pain experienced during childhood. However, individuals rarely complain of facial pain during childhood [3].

Although it is often ascribed to an injury or a headache, facial pain may also be the result of a neurological disorder. Disorders involving both the central nervous system, including the thalamus and somatosensory area of the cerebral cortex, and the peripheral nervous system, including the trigeminal nerve and its branches, may cause facial pain. Trigeminal neuralgia (TN) is a representative disorder characterized by unilateral facial pain due to a functional disorder of the trigeminal nerve. In TN, patients experience characteristic brief, sudden, stabbing, electric-shock-like, and severe episodes of pain [4]. When chronic facial pain lacks the characteristics of other neurological disorders, including TN, a diagnosis of persistent idiopathic facial pain (PIFP) —previously known as atypical facial pain — is made.

We report a case involving an 11-year-old boy with PIFP in whom psychological counseling was effective.

## 2. Case Presentation

An 11-year-old boy visited our outpatient clinic with complaints of persistent right cheek pain. His family history revealed that his father had severe hearing impairment. His medical history showed that he had allergic rhinitis and chronic sinusitis that had been treated until 3 months previously by an otolaryngologist.

**Figure 1:**
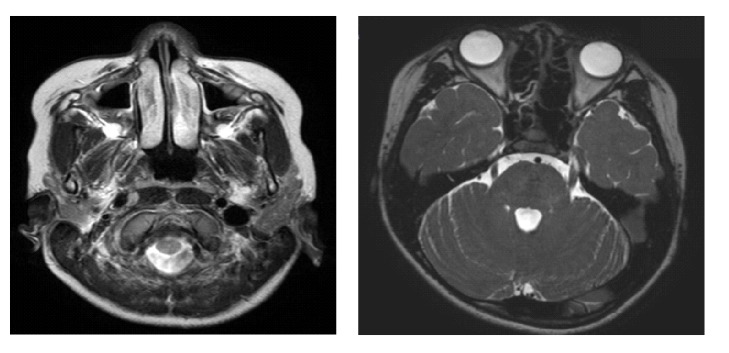
Head imaging study. Head computed tomography showed mucous membrane swelling and fluid collection in both maximal sinuses (left). Brain magnetic resonance image. Vascular compression of the trigeminal nerve was not observed (right).

The patient initially visited our outpatient clinic with complaints of high fever, sore throat, and comorbid right cheek pain and mild swelling 6 weeks previously. A checkup at a dental clinic performed on the day before this initial visit revealed no abnormal findings. He showed clinical symptoms of streptococcal pharyngitis. A rapid antigen test for group A streptococcal infection showed positive results. The patient was diagnosed with streptococcal infection. Facial cellulitis was also suspected and treatment with amoxicillin helped improve symptoms. However, facial pain recurred within 4 weeks of the initial visit. The patient had mild tenderness and swelling of the right cheek. Head computed tomography revealed mild mucous membrane swelling and effusion in both sinuses (Figure 1 left). Because recurrence of cellulitis with sinusitis was suspected, cefditoren pivoxil treatment was initiated. However, because the pain persisted, he visited our department.

Physical examination revealed no abnormal findings except right cheek tenderness in the area that corresponded with the region supplied by the second branch of the trigeminal nerve (the maximally nerve). Although marked tenderness was evident, no point with hyperalgesia, where a light touch elicited severe pain, was observed. No facial paralysis or oral disorders were observed. Blood examination revealed no abnormal findings. Recurrence of sinusitis was suspected. Based on the physical examination and laboratory tests, the patient was clinically diagnosed with TN. Subsequently, oral clarithromycin administration was initiated for sinusitis that might have caused or exacerbated TN. However, administration of clarithromycin for 1 week was not effective for his facial pain. Brain magnetic resonance imaging (MRI) revealed no neurovascular compression (Figure 1 right), which ruled out idiopathic, classical TN. During this time, we interviewed the patient on the nature of the right cheek pain. The patient described the pain as persistent, nagging, and dull in nature, which was completely different from the characteristics of pain associated with TN. Furthermore, trigger maneuvers failed to evoke pain. These evaluations excluded TN, and, thus, PIFP was diagnosed in week 2. Low dose of oral anticonvulsant carbamazepine (50 mg, twice a day) was initiated but was ceased due to general fatigue after the first administration.

Although the patient had been previously cheerful and greeted us when entering the examination room, he became gradually emotionless with headache and nausea in week 5. In addition, feeding difficulties and numbness in the arms occurred. An orthostatic tolerance test revealed no positive findings for orthostatic dysregulation. It became difficult for the patient to attend school in week 6. Because various somatic symptoms developed in addition to PIFP, psychological factors were suspected to be pertinent in the etiology of PIFP. During a detailed medical interview with the patient and his mother, several problems were revealed: the patient loved swimming but his swimming record had plateaued after fixing his swimming form even though he practiced vigorously at a top-class swimming club team. Moreover, because of a recent finger injury, he could not practice as intensely as he wanted; therefore, his competitive ability as a swimmer deteriorated. Furthermore, in early adolescence, the patient had difficulties in communication and his relationship with his father was strained due to the father's hearing impairment. Because these suggested that the circumstances surrounding him might have led to somatoform disorders, psychological counseling was ordered in week 6.

As the patient faced, understood, and tolerated his psychological stress through counseling and psychotherapy twice a week, he gradually became expressive, worked up his appetite, and could attend school in week 10. Although sinusitis recurred at week 29, no facial pain developed. The patient received psychological counseling twice or thrice a month by this time. After 8 months, the frequency of counseling was reduced to once in 2 months. During this period, the patient's voice changed and became deeper at puberty. After confirming that facial pain as well as general malaise did not occur, even when the patient experienced distressing events, such as terminal examinations, counseling was ceased after 1 year and 8 months. After 3 years, the patient went on to high school and currently attends school cheerfully without any complaints and has resumed swimming.

## 3. Discussion

**Figure 2:**
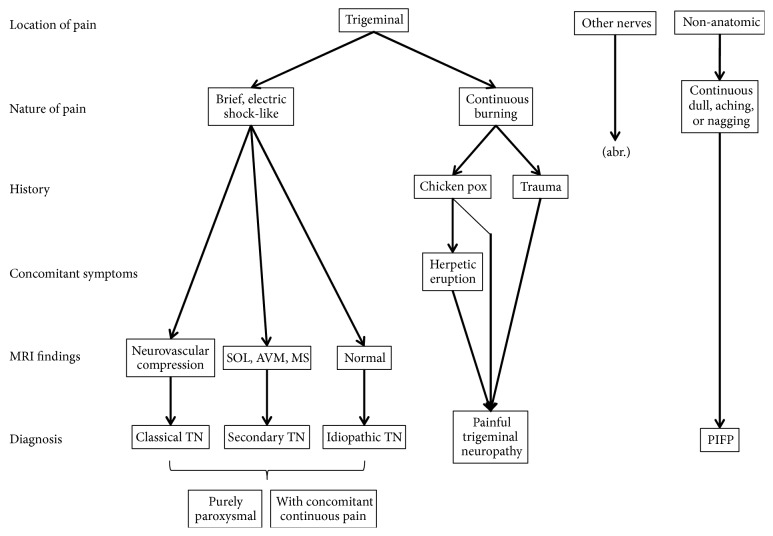
Diagnostic flow chart of facial pain. After injury or dental origin is excluded, advance the diagnosis following the chart. All types of TN have purely paroxysmal and with concomitant continuous pain subtypes. In our case, because we were constrained by the location of the pain (limited to the second branch of the trigeminal nerve), and the characteristics of PIFP are poorly localized, a temporary diagnosis of TN was made. However, as this chart shows, the nature of pain is also important, and enough attention should be paid to pain characteristics as well as its location. Regarding the location in our patient, we may have overlooked pain in other areas. AVM stands for arteriovenous malformation, MS stands for multiple sclerosis, PIFP stands for persistent idiopathic facial pain, SOL stands for solid occupied lesion, and TN stands for trigeminal neuralgia.

After ruling out injury, facial pain may be neurological, vascular, or dental in origin. In this pediatric case of facial pain, dental origin was excluded early. The painful area corresponded with the region supplied by the maximally nerve; this suggested TN. TN during childhood is rare and occurs in only 1.5% of patients [5]. As MRI revealed no neurovascular compression, classical TN, including “classical TN with concomitant continuous pain” was excluded, according to the International Classification of Headache Disorders 3 [6]. In addition, as the patient had no underlying disease that might cause TN such as multiple sclerosis or a brain tumor, secondary TN was also excluded. Furthermore, as the patient had no history of head trauma or surgery and showed no symptoms of herpes zoster, painful trigeminal neuropathy was excluded. Subsequently, “TN attributed to other causes”, “idiopathic TN with concomitant continuous pain” in which no neurovascular compression is observed (previously called atypical TN), and PIFP should be considered as differential diagnoses. The former two are characterized by persistent facial pain in the affected area and should fulfill the diagnostic criteria of classical TN, namely, pain with the following characteristics: unilateral facial pain recurring in paroxysmal attacks lasting from a fraction of a second to 2 minutes; pain of severe intensity; pain with an electric shock-like, shooting, stabbing, or sharp quality; pain precipitated by innocuous stimuli to the affected side of the face [6]. In our case, TN was ruled out because facial pain did not fulfill these criteria. Moreover, the characteristics of the patient's pain were consistent with that of PIFP [6], except that the affected area corresponded with the region supplied by the maximally nerve. Pain in other areas may have been overlooked.  Figure 2 shows a schematic outline of the diagnosis of facial pain.

Subsequently, the diagnosis of PIFP was made by exclusion. Although TN is rare during childhood, there is a report describing that the lifetime prevalence of PIFP (0.03%) is far lower than that of TN (0.3%) [7]. Therefore, pediatric PIFP would be very rare. According to the PIFP criteria [6], patients with PIFP present with high levels of psychiatric comorbidity and psychosocial disability. Taking these together, PIFP in the patient was suspected to be due to somatization of psychological stress.

There is no specific treatment for PIFP. A multidisciplinary biopsychosocial approach with the use of antidepressant and antiepileptic drugs and psychological counseling helps relieve pain [8, 9]. In our case, the patient was obliged to stop carbamazepine because of general fatigue after the first administration. Considering that the patient was under psychological stress, complaints after administration might be attributed to psychological refusal of medical intervention.

Psychological counseling revealed that the patient in his early adolescence had little difficulty in school life but had some problems in his out-of-school life. One was the conflict with his father, which is common in preadolescent boys. However, his father's hearing impairment led to communication difficulties and might have complicated the situation. In addition, although swimming was given considerable importance by the patient, the gap between reality and expectations as a competitive swimmer had widened. Psychological counseling also revealed that he liked reading books and had an excellent understanding of this causal relationship and insight of his situation. Due to these advantages, he understood his position and controlled his own emotion. However, with too much consideration for others, he behaved older than his age, which might have exacerbated psychological stress.

Sand-play therapy and psychological counseling with listening attitude gradually enabled the patient to express his emotion. Appetite loss was first improved. Headache and nausea consecutively disappeared. Lastly, facial pain was gradually improved in accordance with the repetitive counseling. These suggest that somatization due to psychological stress underlies the pathogenic mechanism of PIFP in our case. PIFP might occur as somatization in the patient with overadaptation tendency, triggered by out-of-school problems on the background of the vague sense of unease characteristic to preadolescence. Expression of self-consciousness that the patient acquired through psychological therapeutic support might produce improvements.

The importance of the psychological perspectives of chronic pain has been underscored [10]. When encountering a pediatric patient suspected of having PIFP, one should not only determine the cause of facial pain but also consider the psychological problems that the patient might have.
